# An Army of Women

**Published:** 1887-08-20

**Authors:** Honnor Morten


### AN ARMY OF WOMEN.

Not Amazons we, yet an army of women,
Trained to obey, to daily face death;
Seeking not fame, nor fortune, nor glory,
Stretching no grasping hands forth for a wreath.

Look at our weapons;—pure womanly, surely,
The scissors and forceps, the needles and thread:
Yet hard is our work to watch over the weary,
To render last service to dying and dead.

Look at our uniform;—the cap and the apron,
Symbols that servants and labourers wear.
Our servitude is to the sick and the suffering,
Our labour to stay the sad sob or the tear.

An army whose mission is saving,—not slaying;
Whose aim is to heal instead of to harm.
Let scarlet coats take earthly riches and honour,
We have our reward in hearts that are warm.

Honnor Morten.

